# Role of CD5 signalling for pro-inflammatory Th17 response in multiple sclerosis

**DOI:** 10.1093/brain/awaf268

**Published:** 2025-08-25

**Authors:** Katrin Pape, Nicholas Hanuscheck, Samantha Schmaul, Flores Kneilmann, Georg Bündgen, Beatrice Wasser, Falk Steffen, Sinah Engel, Felix Luessi, Matthias Klein, Toszka Bohn, Tobias Bopp, Stefan Bittner, Frauke Zipp

**Affiliations:** Department of Neurology, Research Center for Immunotherapy (FZI) and Focus Program Translational Neuroscience (FTN), University Medical Center of the Johannes Gutenberg University Mainz, Mainz 55131, Germany; Department of Neurology, Research Center for Immunotherapy (FZI) and Focus Program Translational Neuroscience (FTN), University Medical Center of the Johannes Gutenberg University Mainz, Mainz 55131, Germany; Department of Neurology, Research Center for Immunotherapy (FZI) and Focus Program Translational Neuroscience (FTN), University Medical Center of the Johannes Gutenberg University Mainz, Mainz 55131, Germany; Department of Neurology, Research Center for Immunotherapy (FZI) and Focus Program Translational Neuroscience (FTN), University Medical Center of the Johannes Gutenberg University Mainz, Mainz 55131, Germany; Institute for Immunology, Research Center for Immunotherapy (FZI), University Medical Center of the Johannes Gutenberg University Mainz, Mainz 55131, Germany; Department of Neurology, Research Center for Immunotherapy (FZI) and Focus Program Translational Neuroscience (FTN), University Medical Center of the Johannes Gutenberg University Mainz, Mainz 55131, Germany; Department of Neurology, Research Center for Immunotherapy (FZI) and Focus Program Translational Neuroscience (FTN), University Medical Center of the Johannes Gutenberg University Mainz, Mainz 55131, Germany; Department of Neurology, Research Center for Immunotherapy (FZI) and Focus Program Translational Neuroscience (FTN), University Medical Center of the Johannes Gutenberg University Mainz, Mainz 55131, Germany; Department of Neurology, Research Center for Immunotherapy (FZI) and Focus Program Translational Neuroscience (FTN), University Medical Center of the Johannes Gutenberg University Mainz, Mainz 55131, Germany; Institute for Immunology, Research Center for Immunotherapy (FZI), University Medical Center of the Johannes Gutenberg University Mainz, Mainz 55131, Germany; Institute for Immunology, Research Center for Immunotherapy (FZI), University Medical Center of the Johannes Gutenberg University Mainz, Mainz 55131, Germany; German Cancer Consortium (DKTK), Heidelberg D-69120, Germany; Institute for Immunology, Research Center for Immunotherapy (FZI), University Medical Center of the Johannes Gutenberg University Mainz, Mainz 55131, Germany; Department of Neurology, Research Center for Immunotherapy (FZI) and Focus Program Translational Neuroscience (FTN), University Medical Center of the Johannes Gutenberg University Mainz, Mainz 55131, Germany; Department of Neurology, Research Center for Immunotherapy (FZI) and Focus Program Translational Neuroscience (FTN), University Medical Center of the Johannes Gutenberg University Mainz, Mainz 55131, Germany

**Keywords:** multiple sclerosis, Th17 cells, CD5, CK2, immunomodulation

## Abstract

Pro-inflammatory T-helper 17 (Th17) cells are of vital importance in human autoimmune diseases such as multiple sclerosis (MS). Due to differentiation and functional plasticity, Th17 cells are able to produce a variety of pro-inflammatory cytokines such as interleukin (IL)-17A, interferon (IFN)-γ and granulocyte-macrophage colony-stimulating factor (GM-CSF), and modulation of Th17 cell activities represents a desirable tool for disease-modifying treatment. Here, we aimed to understand the role of the surface molecule CD5 and its intracellular interaction partner casein kinase 2 (CK2) in human Th17 effector function as well as their role in multiple sclerosis.

We performed targeted single-cell RNA sequencing from CSF obtained from people with multiple sclerosis and non-inflammatory neurological diseases, and high-sensitivity proteomic analysis of serum and CSF from 114 people with multiple sclerosis by using a proximity extension assay (PEA) together with functional investigations on CD4+ memory T cells differentiated into a Th17-polarized phenotype.

Blockade of CD5 reduced the production of IL-17A, IFN-γ and GM-CSF by Th17-polarized cells without affecting proliferation. In comparison, blockade of its intracellular interaction partner CK2 exerted partly similar effects with a decrease in IL-17A and GM-CSF production but also impaired T cell proliferation. Both blocking agents resulted in a decreased phosphorylation of the downstream signalling molecule STAT3. The CD5 targeting treatment was able to abolish cytotoxic effects caused by Th17-polarized cells. Importantly, transcriptomic and proteomic analysis showed that CD5 expression correlates with an inflammatory immune profile in multiple sclerosis in serum as well as CSF.

Our study highlights the importance of the CD5-CK2-STAT3 signalling axis for inflammatory responses of human Th17-polarized cells. Since in humans CD5 expression correlates with inflammation and cellular injury, targeting the CD5 signalling pathway provides future therapeutic opportunities for—among other diseases—multiple sclerosis.

## Teaser

Pape *et al.* link the CD5-CK2-STAT3 signalling pathway to both inflammation and effector function in multiple sclerosis, using a multi-modal approach that combines functional assays, targeted single-cell RNA sequencing of CSF, and proteomic analyses of CSF and serum.

**See Villar (https://doi.org/10.1093/brain/awaf469) for a scientific commentary on this article.**

## Introduction

T-helper 17 (Th17) cells are a subset of pro-inflammatory CD4+ T-helper (Th) cells and play a major role in many autoimmune disorders. Although they are mainly characterized by the secretion of interleukin-17 (IL-17), Th17 cells display differential plasticity and can shift towards a Th1-like phenotype co-producing IL-17 and interferon-γ (IFN-γ) in chronic inflammatory settings.^1-3^ Importantly, these IFN-γ co-producing Th17 cells are emerging as key players in multiple sclerosis (MS), a chronic autoimmune inflammatory disease of the CNS. For example, they have been found to transmigrate preferentially across the human blood–brain barrier (BBB) and to be enriched in CSF in the early stages of MS.^4,5^ Furthermore, the production of granulocyte-macrophage colony-stimulating factor (GM-CSF) by Th17 cells has been linked to myelin reactivity of Th cells and neuroinflammation.^6,7^ It is thus assumed that Th17 cells co-producing multiple pro-inflammatory cytokines contribute to BBB disruption and neurotoxicity.^8^ Over time, cerebral inflammation and demyelination driven by these autoreactive cells cause progressive neurodegeneration and cumulative disability in young adults.

Interference with pro-inflammatory signalling pathways proved a promising therapeutic strategy in the MS mouse model experimental autoimmune encephalomyelitis (EAE). In these mice, inhibition of the protein kinase CK2 (formerly casein kinase 2) led to suppression of Th17 cell development and amelioration of EAE.^9^ The CK2 intracellular serine/threonine kinase interacts with the cytoplasmic domain of the surface receptor CD5, a molecule involved in both positive and negative regulation of T cell receptor signalling,^10^ and T cell-specific deficiency in CD5-CK2 binding and activation sites resulted in EAE resistance.^11^ Alternative co-stimulation of Th cells via CD5 is supposed to be a stabilizing factor in Th17 differentiation.^12^ In addition, phosphorylation of a downstream effector molecule of CD5, namely signal transducer and activator of transcription 3 (STAT3), is a key checkpoint in Th17-driven autoimmunity, promoting the transcription factor retinoic acid receptor-related orphan receptor gamma t (RORγt) and expression of the IL-23 receptor.^13^ Collectively, these data point towards a role for CD5 in Th17-driven autoimmune diseases. However, studies investigating this possibility have largely been conducted in mouse models, in which the properties of Th cell subsets are known to differ significantly from humans.^14^

Here, we aimed to elucidate the CD5-CK2-STAT3 axis in human Th17 function. We demonstrate that blockade of CD5 impairs production of pro-inflammatory cytokines by Th17-polarized cells and attenuates their cytotoxic effects. Importantly, we provide evidence for the correlation of CD5-triggered signalling with inflammatory profiles in MS.

## Materials and methods

### Patients and controls

This study was approved by the local ethical review board (numbers 837.019.10 and 2019-14758_1) with all participants having provided informed and written consent in accordance with the Declaration of Helsinki. CSF samples were generated from lumbar punctures in routine diagnostic settings and used for further analysis. Proteome data derived from 114 patients undergoing initial diagnostic procedures for possible MS at the Department of Neurology of the University Medical Center Mainz were included. Inclusion criteria were: (i) receiving a diagnosis of clinically isolated syndrome (CIS) or relapsing-remitting MS (RRMS) according to the 2017 revised McDonald criteria^15^; (ii) first episode of neurological symptoms suggestive of MS no earlier than 5 years before sample collection; (iii) no disease-modifying or steroid treatment prior to sample collection; (iv) aged no older than 50 years at the time point of sample collection; (v) availability of cranial MRI within 3 months of sample collection; and (vi) availability of paired cell-free CSF and serum samples collected at the time of diagnosis and stored at our biobank. Single-cell RNA sequencing (scRNA-seq) was performed with CSF from three patients with treatment-naive RRMS and three control patients with non-inflammatory neurological diseases (NIND). Patient characteristics are provided in Supplementary Table 1 (proteome cohort) and Supplementary Table 2 (scRNA-seq cohort).

### Proteomics

Proteins were measured with the Olink® Inflammation panel using proximity extension assay (PEA) technology, a high-throughput multiplex proteomic immunoassay.^16^ For one analysis, the protein neurofilament light polypeptide (NEFL) from the Olink Neuro Exploratory panel was included. Proteomic analyses were performed by Olink® after sample preparation according to the manufacturer’s protocol (http://www.olink.com). All assays included four internal controls to monitor the performance and reliability of the assay. This includes two incubation control proteins (phycoerythrin and green fluorescent protein), an extension control consisting of immunoglobulin G (IgG) antibodies conjugated with a matching oligo pair and a detection control with a synthetic double-stranded DNA. Samples with extremely high deviation from plate median [±0.6 normalized protein expression (NPX])] were treated as missing data. In addition, external controls including inter-plate, negative and inter-batch controls were used to determine potential issues in assay quality and errors in handling along with general normalization of batch effects. Markers were filtered based on their call rate (i.e. proportion of samples with concentrations above the lower limit of detection). Proteins with a call rate below 25% were excluded (8 proteins in serum and 33 proteins in CSF). Details on the proteome panel are provided in Supplementary Table 3. Distributions of protein levels were compared between groups using Wilcoxon rank-sum test with Holm–Bonferroni correction for multiple testing.

### Single-cell RNA sequencing

Capturing single cells and cDNA synthesis was performed using the BD Rhapsody system (BD Biosciences; single-cell capture and cDNA synthesis with the BD Rhapsody single-cell analysis system; Doc ID: 210966 Rev. 1.0). The targeted human immune response panel (BD, including a panel of 399 markers with significant impact on neuroimmunology) together with custom selected primers for the detection of additional gene expression was used for library preparation (464 markers in total). The mRNA libraries were prepared according to the manufacturer’s instructions (mRNA targeted and sample tag library preparation with BD single-cell multiplexing kit; Doc ID 214062 Rev. 1.0). Upon purifying each library, the concentration of libraries was assessed by Qubit 2.0 using the DNA high-sensitivity kit (Invitrogen) and average library size was determined on a bioanalyser (Agilent) using a high-sensitivity DNA chip (Agilent). The final sequencing library was prepared by dilution and pooling the targeted mRNA library following the recommendations of the sequencing preparation sheet provided by BD. The libraries were sequenced on a HiSeq2500 (Illumina) using a HiSeq PE Rapid Cluster Kit v.2 (PE-402-4002) and combining three HiSeq Rapid SBS Kits v.2 (50 cycles). We obtained a mean of 3443 cells per sample (min. 2507 cells, max. 4107 cells, median 3676 cells/sample).

Details for scRNA-seq data analysis are provided in the Supplementary material, Methods.

### Human Th17 cells

Memory CD4+ T cells were magnetically isolated from peripheral blood mononuclear cells (PBMCs) following gradient centrifugation and skewed into effector Th17 cells as previously described.^17^ For blocking experiments, cells were treated with 10 µg/ml anti-CD5 or matched unspecific IgG1 antibody, 10 μM CX4945 or dimethyl sulfoxide (DMSO) as a control condition for CX4945. Flow cytometry and intracellular cytokine staining were performed as previously described.^4^ To determine cell proliferation, T cells were labelled with 2.5 µM carboxyfluorescein succinimidyl ester as described.^9^ Proliferation was measured on Day 3 using flow cytometry. For quantification, the division index was calculated using the average number of divisions for all cells in the original culture.^18^ Staining for phosphorylated STAT3 (pSTAT3) was performed as previously described using the BD Phosflow™ kit.^9^ The positive control was stimulated with phorbol-12-myristate-13-acetate (PMA) (1:500) and 20 ng/ml IL-23. The pSTAT3-antibodies were used as indicated above. For flow cytometry, the following fluorescent dye-labelled antibodies were used: anti-CD3 Alexa Fluor (AF)700 (clone UCHT1), anti-CD4 peridinin-chlorophyll-protein complex (PerCP) (clone SK3), anti-CD5 v450 (clone UCHT2) and corresponding isotype control, anti-CD14 fluorescein isothiocyanate (FITC) (clone M5E2), anti-IL-17A FITC (clone BL168), anti-IFN-γ phycoerythrin-cyanine 7 (PE-Cy7) (clone B27), anti-GM-CSF PE (clone BVD2-21C11), anti-pSTAT3 S727 AF647 (clone 49/p-Stat3) and corresponding isotype control, anti-pSTAT3 Y705 PE (clone 4/P-STAT3) and corresponding isotype control. Flow-cytometric experiments were performed on a BD Canto II and analysed using BD FACSDiva software and FlowJo software (Tree Star, Inc; Ashland, OR, USA.).

### Co-culture of human Th17 cells with a human neural cell line

H9-derived human neural stem cells (H9 hNSCs, Gibco) were cultured as previously published.^19^ For co-culture, Th17-polarized cells were restimulated with CD3/CD28 Dynabeads (ThermoFisher) in a T cell to bead ratio of 1:10 overnight. Afterwards, cells were washed, recounted and stained with 4-(5)-(((4-chloromethyl)benzoyl)amino)tetramethylrhodamine (CMTMR) cell tracker dye orange (Invitrogen) 1:1000 in XVivo Medium (15 min, 37°C). In co-cultures with a cytotoxic effect of Th17-polarized cells, comparative analysis was performed with Th17-polarized cells pretreated with CD5 blockade. After 72 h of co-culture, cells were fixed with 4% paraformaldehyde for 20 min at room temperature and stained via immunocytochemistry.

### Immunocytochemistry

Immunocytochemistry of human co-cultures was performed as previously published.^19^ After blocking, cells were incubated with anti-Nestin primary antibody (1:500, R&D) overnight at 4°C. After washing with PBS, secondary antibody incubation was performed for 2 h with AlexaFluor-647-conjugated anti-mouse secondary antibodies (1:500, Life Technologies) and 4′,6-diamidino-2-phenylindole (DAPI) (1:10 000, Invitrogen). Coverslips were analysed using a Leica TCS SP8 equipped with a DM 6000CS stand using a 63× objective (Leica, numerical aperture 1.4) and a resolution of 1024 × 1024 pixels. Fields of view were selected at random and three to five *z*-stacks were acquired per coverslip. Quantification of the remaining H9 cells after co-culture with Th17 cells was performed in a blinded fashion using ImageJ (NIH; Bethesda, Maryland, USA).

### Quantification of cytokines

Absolute values of cytokines IL-17A, IFN-γ and GM-CSF were measured using an Ella™ automated multiplex ELISA platform. A multiplex cytokine assay was performed according to the manufacturer’s instructions.

### Statistical analyses

Data analysis was performed using GraphPad Prism software (version 7.00 for Windows, GraphPad Software, La Jolla, CA, USA). Data are shown as the mean ± standard error of the mean (SEM) unless otherwise indicated. Statistical analysis of targeted scRNA-seq data was performed in R 4.3.0, and statistical analysis of Olink® proteomic data was performed in R 4.4.2 [R Core Team (2024). _R: A Language and Environment for Statistical Computing_. R Foundation for Statistical Computing, Vienna, Austria. https://www.R-project.org/], using log_2_ transformed protein measurements (NPX). Significance was set at *P < 0.05.

## Results

### Blockade of both CD5 and CK2 impairs Th17 function

**Figure 1:**
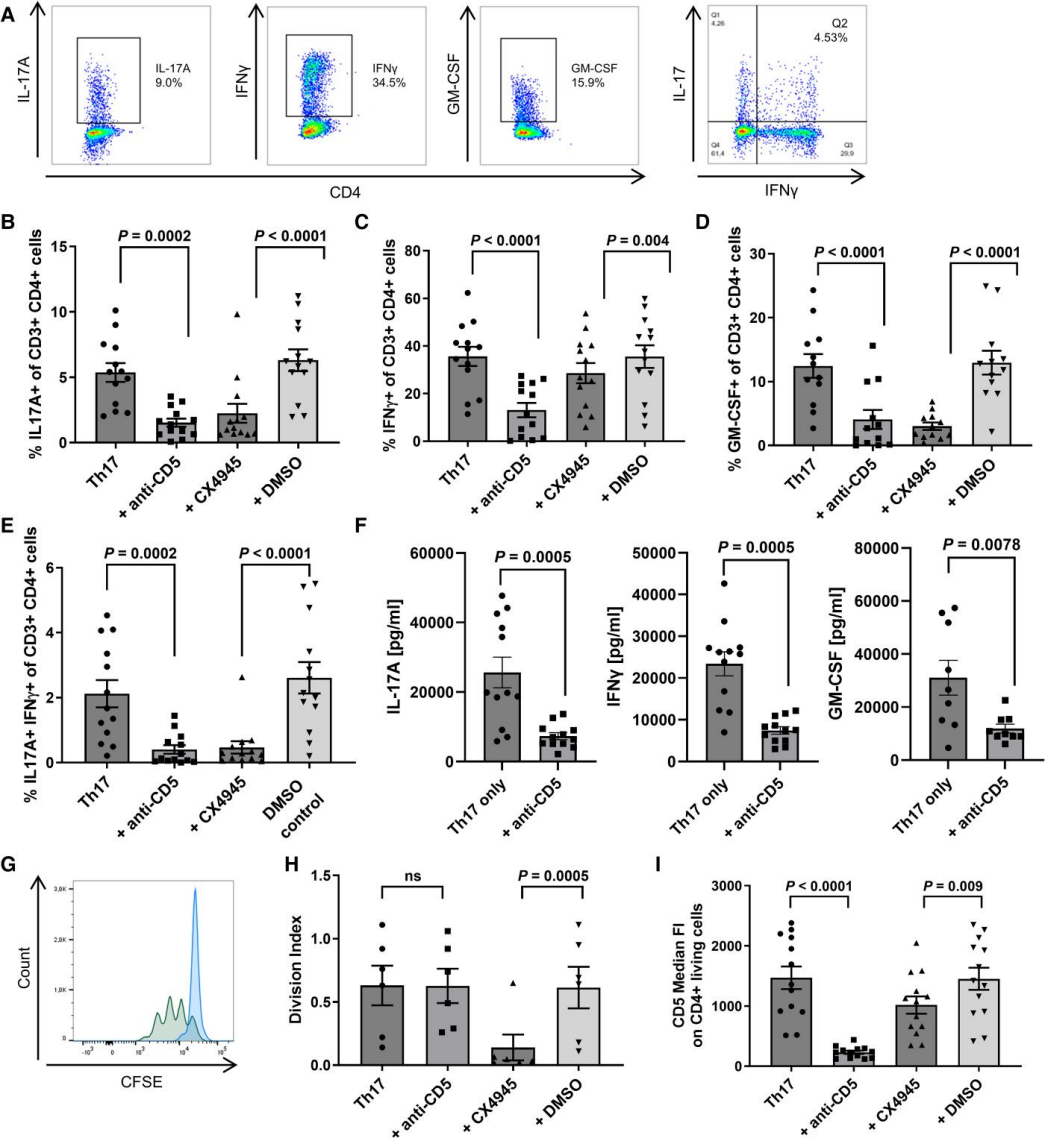
**Effects of CD5 and CK2 blockade on Th17-polarized T cells.** (**A**) Flow cytometric data of a representative intracellular cytokine staining for IL-17, IFNγ, IL-17/IFNγ-double positive cells and GM-CSF. (**B**–**E**) Treatment of Th17-polarized T cells with CD5 blocking antibody or the CK2 inhibitor CX4945 leads to the reduced production of pro-inflammatory cytokines IL-17, IFN-γ and GM-CSF after 7 days of culture. DMSO treatment serves as a control condition for the CK2 inhibitor CX4945. Experiments were conducted with *n* = 13 cultures for IL-17 and IFN-γ, and with *n* = 12 cultures for GM-CSF. (**F**) Absolute quantification of cytokines IL-17A, IFN-γ and GM-CSF in Th17 cell culture supernatants with and without CD5 blocking antibody. Experiments were performed with *n* = 12 cultures. (**G**) Representative histogram for CFSE staining indicating cell proliferation after 3 days of culture. Green = Th17 control condition, blue = CX4945 treatment. As the same total amount of cells was used in both conditions, more proliferation leads to lower cell counts per division subset. (**H**) Treatment with CX4945, but not with anti-CD5, leads to reduced T cell proliferation in culture. The division index is calculated using the average number of divisions for all cells in the original culture. (**I**) CD5 expression on the cell surface on Day 6 of culture was reduced significantly after both CD5 blockade and, to a lesser extent, CK2 inhibition with CX4945. Statistical analysis was performed by two-way ANOVA with Tukey correction for multiple comparisons (**B**–**E**, **G** and **H**) or Wilcoxon test (**F**). Exact *P*-values are indicated between Th17 only and anti-CD5, and between CX4945 and DMSO control. Each culture represents a unique human donor. CFSE = carboxyfluorescein succinimidyl ester; DMSO = dimethyl sulfoxide; GM-CSF = granulocyte-macrophage colony-stimulating factor; IFN = interferon; IL = interleukin; Th = T helper.

To unravel the role of CD5 in human Th17 differentiation, we cultured CD4+ memory T cells under Th17-polarizing conditions for 6 days in the presence or absence of the anti-CD5 antibody as well as the CD5-dependent signalling pathway kinase CK2 inhibitor CX4945. As previously shown by us, the protocol used here resulted in expression of the Th17 transcription factor RORγt.^20^ Th17 polarization led to the co-production of IL-17A, IFN-γ and GM-CSF. In the presence of the anti-CD5 antibody, production of all pro-inflammatory cytokines was significantly reduced regarding both percentage of cytokine-producing cells and absolute cytokine values (Fig. 1A–F and Supplementary Fig. 1). Treatment with CX4945 led to decreased production of IL-17A and GM-CSF without a significant effect on IFN-γ. While T cells treated with CX4945 showed reduced proliferative capacity, there was no change in proliferation upon addition of the anti-CD5 antibody (Fig. 1G and H). Surface expression of CD5 on Day 6 was decreased in the presence of the anti-CD5 antibody and, to a lesser extent, in the presence of CX4945 (Fig. 1I). Thus, blockade of both CD5 and CK2 impaired Th17 functionality, although with differential effects on cell proliferation.

### Downstream signalling of CD5 and CK2 occurs via STAT3

**Figure 2:**
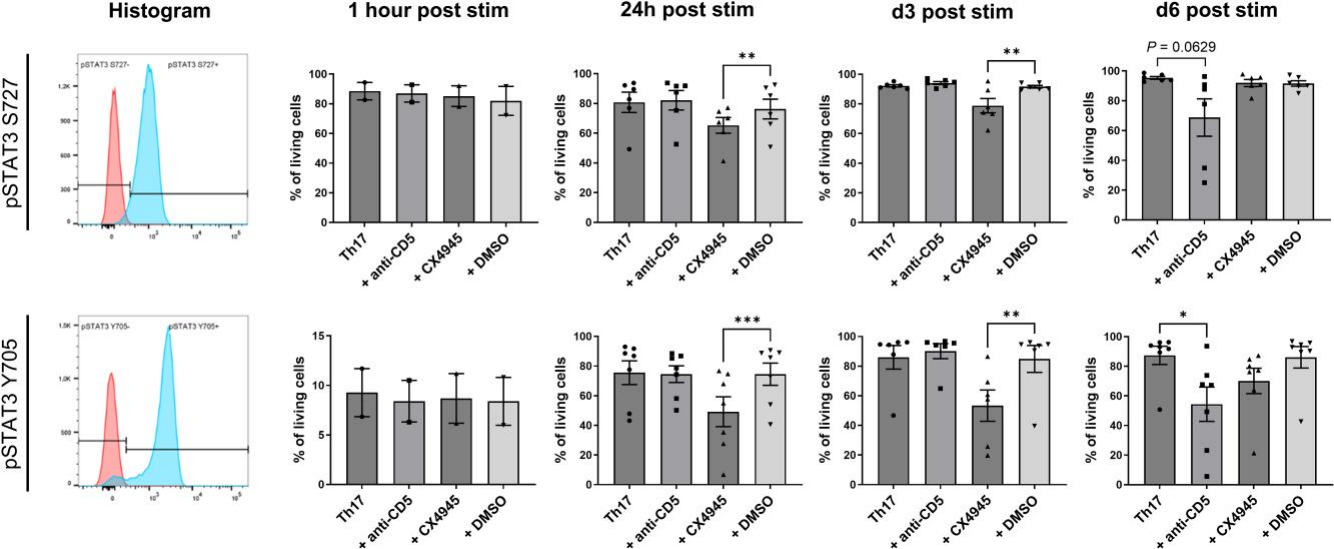
**Inhibition of Th17 polarization is mediated by phosphorylation of STAT3**. In the *left* column, representative histograms are shown for staining of the two main STAT3 phosphorylation sites S272 (*top row*) and Y705 (*bottom row*). Red = isotype control, blue = positive control (stimulation with PMA and IL-23). One hour after stimulation (stim), no difference was observed between the different conditions. CX4945 treatment led to significantly reduced STAT3 phosphorylation after 24 h and 3 days, while CD5 blockade led to significantly reduced STAT3 phosphorylation after 6 days at the S727 phosphorylation site and a trend towards the same effect at the Y705 phosphorylation site. Data were obtained from two independent experiments for 1 h post-stimulation and at least six independent experiments for all other time points. Statistical analysis was done using a two-way ANOVA with Tukey correction for multiple comparisons. *P < 0.05, **P < 0.01 and ***P < 0.001. Each experiment represents a unique human donor. IL = interleukin; PMA = phorbol-12-myristate-13-acetate; STAT = signal transducer and activator of transcription; Th = T helper.

To assess downstream pathways of the different blocking agents, we chose to analyse STAT3 phosphorylation as a key signalling molecule contributing to Th17 cell differentiation. We performed intracellular phosphorylation staining for both phosphorylation sites important for STAT3 activation, Ser727 (S727) and Tyr705 (Y705), and analysed cells at different time points by flow cytometry. In line with their impact on cytokine production, both inhibition of CK2 and blockade of CD5 decreased STAT3 phosphorylation at both S727 and Y705, although they showed different kinetics with a maximum effect of CX4945 after 3 days compared with 6 days for treatment with the anti-CD5 antibody (Fig. 2).

### Blockade of CD5 signalling axis ameliorates Th17-induced cytotoxicity

**Figure 3:**
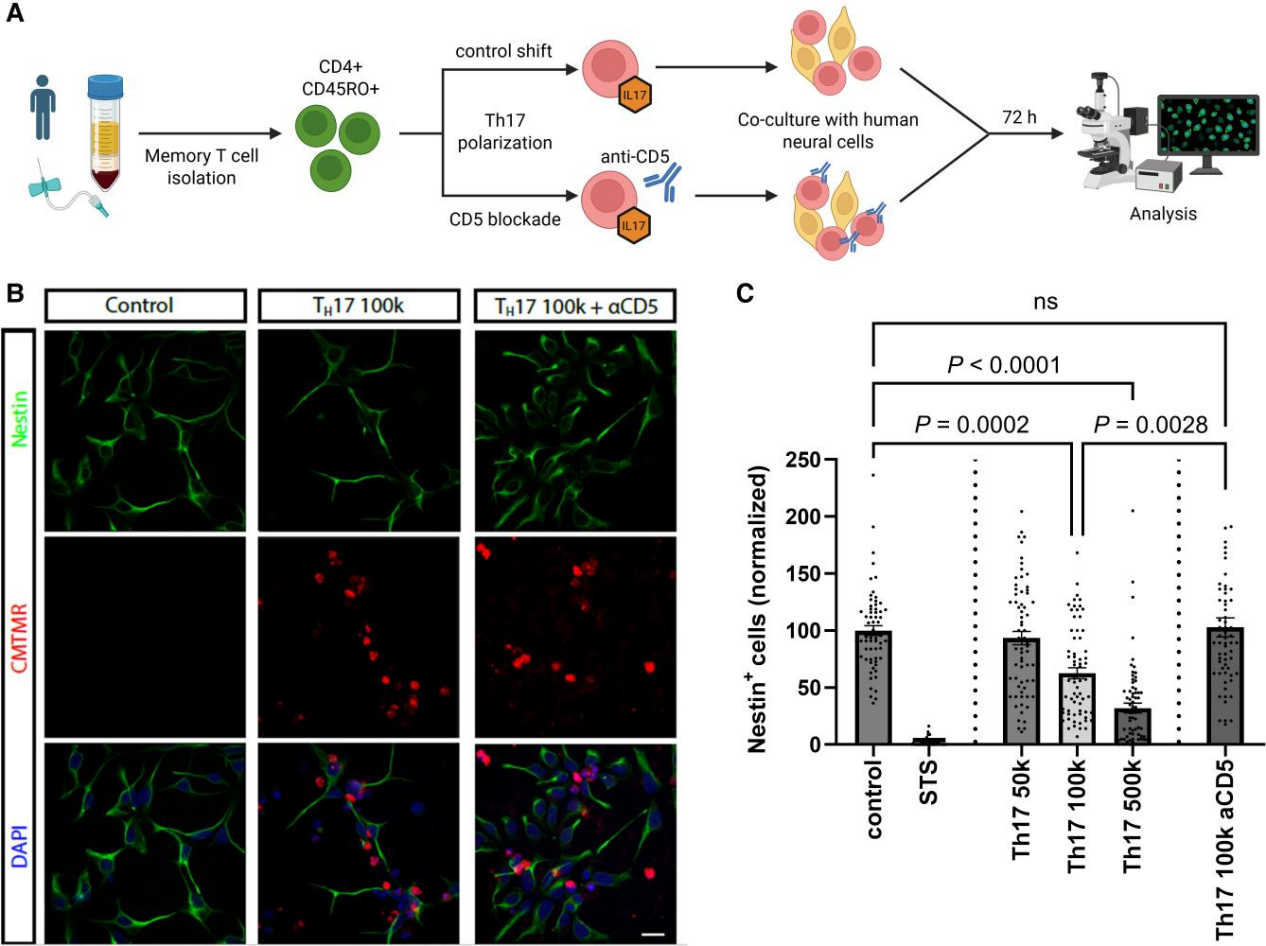
**Th17-induced cytotoxicity can be rescued by CD5 blockade**. (**A**) Experimental paradigm for co-culturing of Th17 cells and human neural cells. CD4+ CD45RO+ T cells from healthy donors were cultured under Th17-polarizing conditions with or without CD5-blocking antibody for 7 days. Then, cells were co-cultured with target cells for 72 h. Created in BioRender. Pape, K. (2025) https://BioRender.com/v18p933. (**B**) Representative confocal images of human neural cells after co-culture showing a reduction of Nestin+ NSC (green) after engagement with Th17 cells (labelled with CMTMR, red) which was rescued by CD5 blockade. Nuclei labelled with DAPI (blue). Scale bar = 20 µm. (**C**) Quantification of Th17-mediated damage on target cells. The kinase inhibitor Staurosporin (STS, 4 µM) was used as a positive control for cell death. Statistical analysis was performed by Kruskal–Wallis testing and Dunn’s correction for multiple comparisons. Pooled data from five independent experiments. Each experiment represents a unique human donor. CMTMR = 4-(5)-(((4-chloromethyl)benzoyl)amino)tetramethylrhodamine; DAPI = 4',6-diamidino-2-phenylindole; NSC = neural stem cells; Th = T helper.

Th17 cells are involved in CNS inflammation and brain damage. This may occur not only via the production of pro-inflammatory cytokines, but also by cytotoxic effects. To assess the impact of modulation of T cell function on cytotoxicity, we established an *in vitro* co-culture model with human neural cells vulnerable to Th17-mediated effector function and thereby enabling comparison of Th17-modulating approaches (Fig. 3A). This enabled us to investigate human Th17-polarized T cells inducing cytotoxicity in an major histocompatibility complex (MHC)-II/antigen-independent manner towards neural cells as an approximate model for human pathology. In fact, Th17-polarized cells led to significant cellular damage as shown by the reduction of target cells; this effect was abolished upon anti-CD5 treatment (Fig. 3B and C and Supplementary Fig. 2).

### Increased activity and homing properties of CD5+ T-helper cells in MS compared with controls

**Figure 4:**
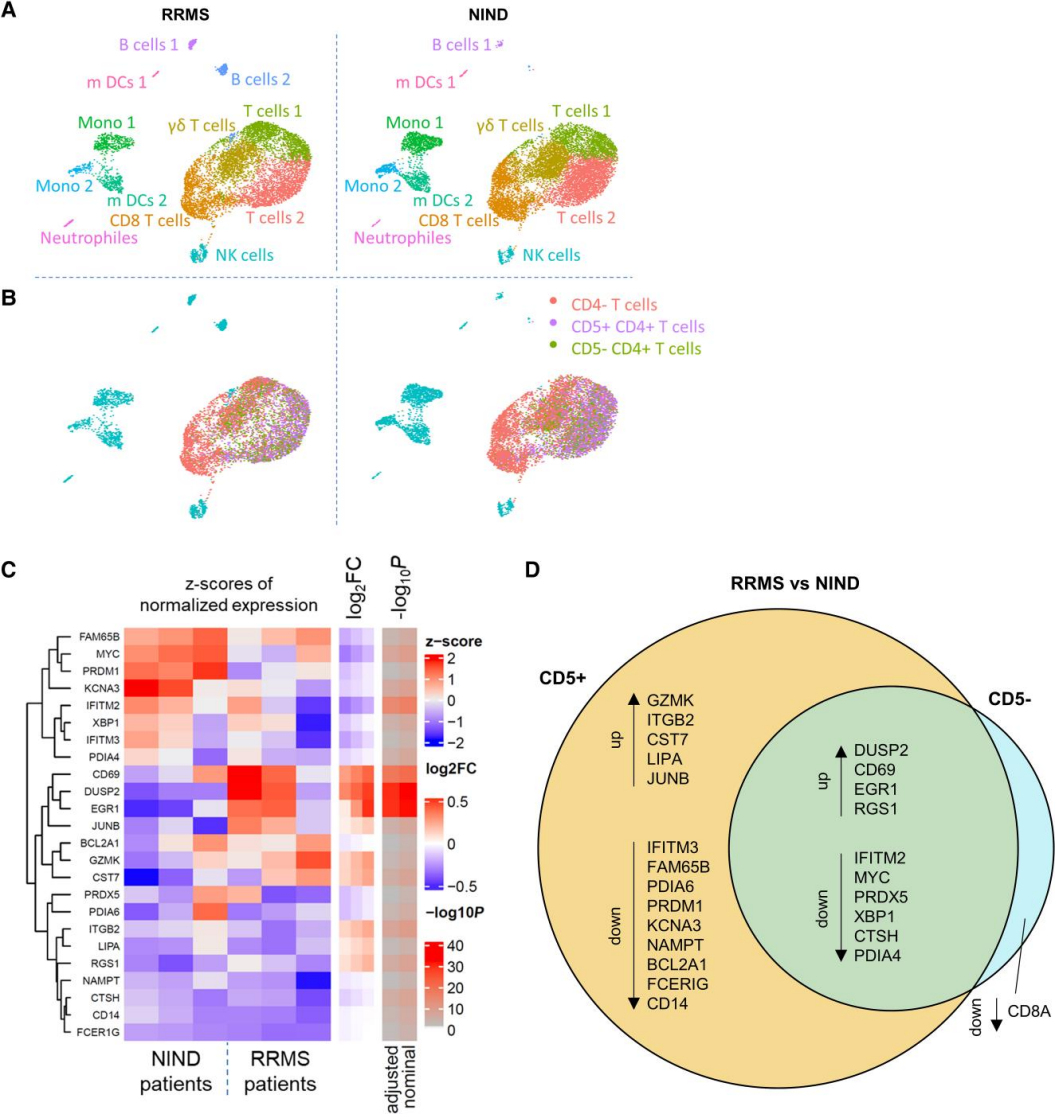
**Analysis of T cells in CSF by targeted single-cell RNA sequencing (scRNA-seq)**. (**A**) CSF cells after doublet removal are shown as UMAP visualization of 11 principle components. The panel on the *left* displays samples from patients with relapsing-remitting multiple sclerosis (RRMS, *n* = 3), and on the *right*, control patients with non-inflammatory neurological diseases (NIND, *n* = 3). Colour-coded Leiden clustering of cells annotated by cell types. The annotation of ScType was manually changed from ‘macrophages’ to ‘mono(cytes)’, from ‘CD8+ NKT-like cells’ to ‘CD8 T cells’ and from the ‘immune system cells’ as well as the ‘memory CD8+ T cells’ to ‘T cells’. Additionally both ‘B cells’ clusters were annotated as ‘memory B cells’ by ScType. (**B**) Subsets of cells used for differential gene expression testing in (*left*) RRMS and (*right*) NIND. Teal = cells not confirmed to be T cells (by cluster annotation, in the case of scType’s ‘immune system cells’ any CD3 gene expression had to be detected to count as a T cell). For cells to count as CD4 or CD5 positive they had to reach a threshold of 0.5 for the log-transformed normalized expression. (**C**) Differential gene expression (DGE) testing RRMS versus NIND inside the subset of CD5+ CD4+ T cells. Heat map of expression in the CD5+ subset for significantly regulated genes. *Left* side shows hierarchical clustering of genes. Log_2_ fold changes are shown as lower bound of confidence interval (*left*), point estimate (*middle*) and upper bound (*right*). *P*-values are shown in a column for Bonferroni-adjusted and one for nominal. Image created with R package ComplexHeatmap. (**D**) Venn Diagram of DGE results RRMS versus NIND (mixed-effect MAST) in both CD5+ and CD5− subsets of CD4+ T cells: names of common and unique significantly regulated genes (Bonferroni adjusted *P* < 0.05) are indicated, sorted by fold change. DGE = differential gene expression; MAST = model-based analysis of single-cell transcriptomics; NKT = natural killer T cells; UMAP = Uniform Manifold Approximation and Projection.

While these results provided proof-of-concept for a functional impact of CD5 on neuroinflammation in humans, we next performed targeted scRNA-seq in CSF samples from patients with untreated RRMS and with NIND serving as controls. First, we identified cell populations by unbiased graph-based clustering and annotation based on expression of relevant marker genes (Fig. 4A). In addition, we classified CD4+ T cells into CD5+ and CD5− subsets (Fig. 4B). In order to count as CD4+ or CD5+, cells had to reach a threshold of 0.5 for the log-transformed normalized expression. Next, we assessed differentially expressed genes between CD5+ CD4+ T cells from people with MS and from NIND. Analysis of differential gene expression (DGE) revealed upregulation of markers involved in the regulation of cell differentiation and activation (Fig. 4C, Supplementary Fig. 3A and Supplementary Table 4). By comparing those results to DGE in CD5− CD4+ T cells from people with MS and NIND (Supplementary Fig. 3B and Supplementary Table 5), we observed that some markers were increased in people with MS in both T cell subsets, while other markers were increased specifically in the CD5+ subset (Fig. 4D). Those specific markers included, for example, granzyme K (GZMK) and integrin subunit-β 2 (ITGB2), as markers for a brain homing and activation signature in MS compared with NIND.

**Figure 5:**
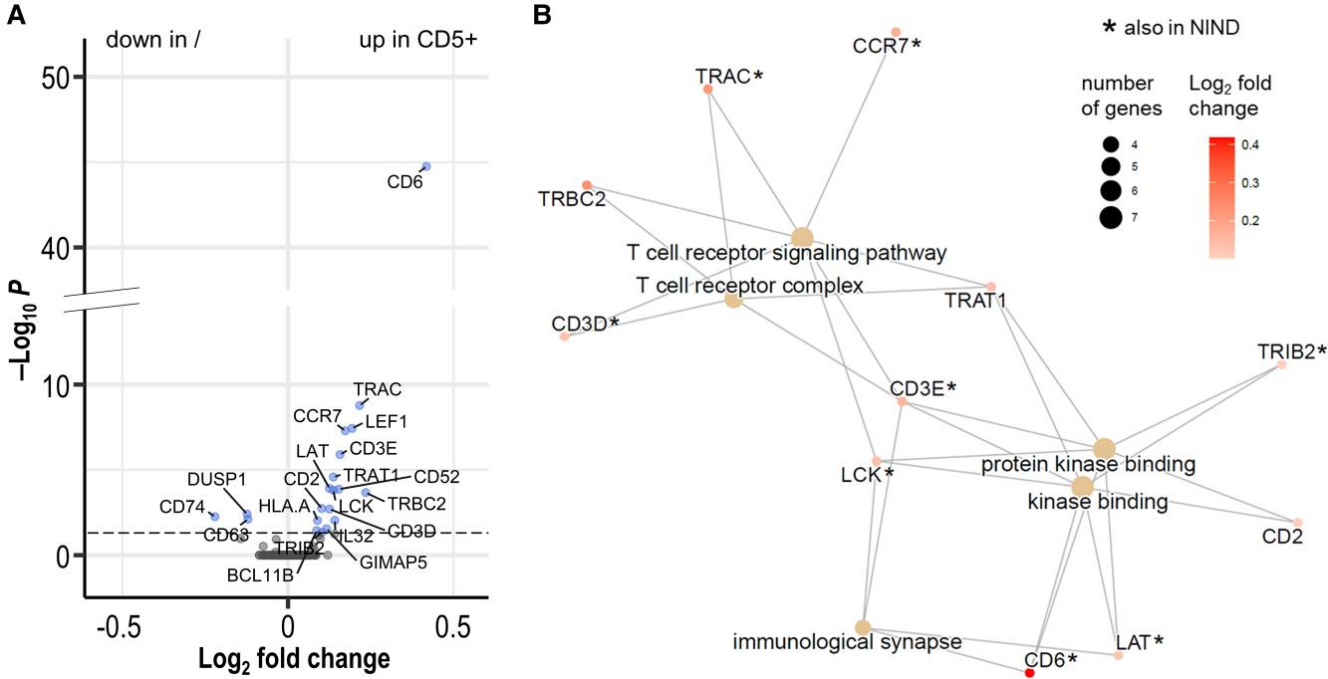
**Comparison of CD5+ and CD5− CD4+ T cells in MS by scRNA-seq**. (**A**) Genes regulated between CD5+ and CD5− T cells in MS. Mixed-effect MAST DGE-testing CD5+ versus CD5− inside the subset of CD4+ T cells of patients with RRMS. Volcano plot of DGE-results. The *x*-axis represents the point estimates for the log_2_ fold changes and the *y*-axis shows the Bonferroni-adjusted *P*-values with the significance threshold of 0.05 marked by a dashed line. Image created with R package EnhancedVolcano. (**B**) Over-representation analysis of all gene ontologies (GO) in significantly upregulated genes. Top 5 enriched GO terms plotted with respective genes coloured by their log_2_ fold change (point estimates). Image created with R package enrichplot. FDR-adjusted *P*-value of ‘immunological synapse’ = 0.0349, which is the highest of the shown terms. Asterisk behind gene symbols means the same gene was significantly upregulated in the same test with samples of NIND patients too. DGE = differential gene expression; FDR = false discovery rate; MAST = model-based analysis of single-cell transcriptomics; MS = multiple sclerosis; NIND = non-inflammatory neurological diseases; RRMS = relapsing-remitting multiple sclerosis; scRNA-seq = single-cell RNA sequencing.

Further, in MS patients, we found upregulation of markers for autoimmune inflammation such as IL-32, for T cell activation such as CD52 and for T cell homing such as CCR7 in CD5+ CD4+ T cells compared with CD5− CD4+ T cells (Fig. 5A and Supplementary Table 6). Gene ontology (GO) term analysis confirmed these results, highlighting the upregulation of T cell signalling and activation pathways in CD5+ CD4+ T cells (Fig. 5B).

### CD5 is linked to neuroinflammation and axonal damage in MS

**Figure 6:**
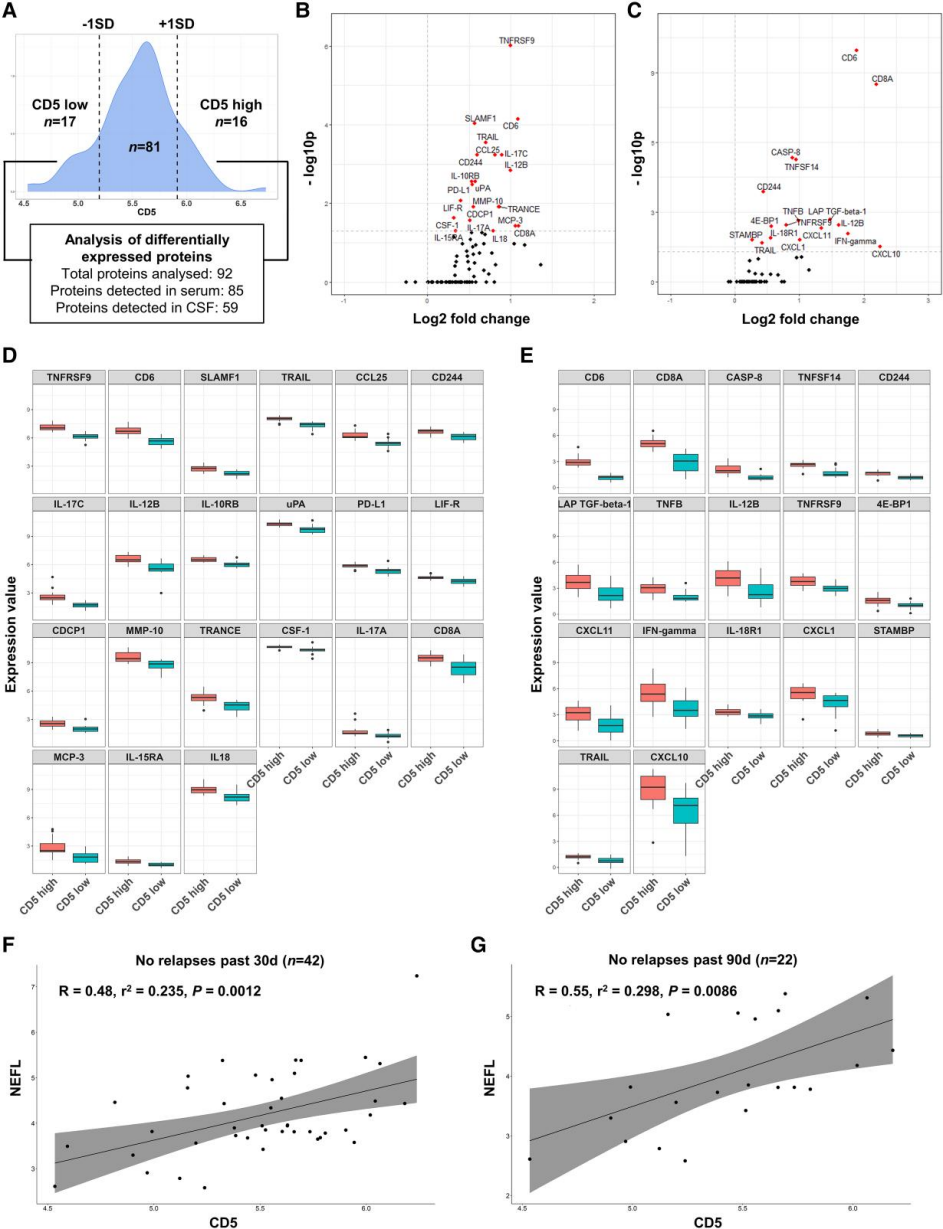
**Analysis of proteins associated with high CD5 expression**. Protein expression values are indicated in a relative quantification measure in Olink’s arbitrary unit called Normalized Protein eXpression (NPX), which is in Log_2_ scale. (**A**) Flow chart of statistical analysis. Groups with high versus low expression of CD5 were defined by mean ± 1 standard deviation, respectively. Example histogram is shown for serum. Serum and CSF were analysed independently. (**B**) Volcano plot showing differentially expressed proteins between CD5 high (*n* = 16) and CD5 low (*n* = 17) groups in serum. Significantly upregulated proteins in the CD5 high group with an adjusted *P*-value < 0.05 are shown in red and labelled. (**C**) Volcano plot showing differentially expressed proteins between CD5 high (*n* = 21) and CD5 low (*n* = 22) groups in CSF. Significantly upregulated proteins in the CD5 high group with an adjusted *P*-value < 0.05 are shown in red and labelled. (**D**) Box plots for all significantly regulated proteins in serum. (**E**) Box plots for all significantly regulated proteins in CSF. (**F**) Correlation between CD5 and neurofilament light (NEFL) in serum using Pearson correlation coefficient, in a subcohort of patients without relapse activity in the past 30 days (*n* = 42). (**G**) Correlation between CD5 and NEFL in a subcohort of patients without relapse activity in the past 90 days (*n* = 22).

Next, in 114 paired serum and CSF samples of people with MS, we applied PEA technology, a high-throughput multiplex proteomic immunoassay,^16^ with a panel of 92 immune-related proteins including CD5 (see Supplementary Table 3 for the complete panel). Based on the distribution of CD5 expression, we defined two subgroups: CD5 high included samples with CD5 expression greater than the mean +1 standard deviation (SD), CD5 low consisted of samples with CD5 expression less than the mean −1 SD (Fig. 6A). Analysis of differential protein expression showed that high CD5 expression is associated with upregulation of proteins involved in pro-inflammatory Th1/Th17 signalling and activation, such as CD137 (TNFRSF9), IL-17C, IL12-B, PD-L1, CD254 (TRANCE), IL-17A or IFN-γ, in both serum and CSF (Fig. 6B–E and Supplementary Tables 7 and 8). In addition, in patients without acute relapse activity, serum CD5 protein expression correlated with neurofilament light as a marker for neuroaxonal damage (Fig. 6F and G) which indicates a link between CD5 expression and neuroaxonal injury.

## Discussion

CD5 is a co-receptor of the T cell receptor (TCR)-CD3 complex and has traditionally been regarded as a negative regulator of TCR signalling and CD4 lineage development.^21,22^ In this study, we show that human Th17-polarized cells co-producing IL-17A, IFN-γ and GM-CSF are mitigated in their pro-inflammatory and cytotoxic function by blockade of CD5. Our data provide evidence that CD5 and CK2 act partly via a common signalling axis in human T cells. Blockade of both molecules reduced secretion of IL-17A and GM-CSF by Th17-polarized cells, and decreased phosphorylation of the downstream agent STAT3. In addition, surface expression of CD5 was reduced significantly upon both inhibitory treatments. While decreased CD5 expression upon CD5 blockade even after 6 days might only reflect the antibody’s direct effect on the surface molecule, the reduction of CD5 expression upon CX4945 treatment underlines the reciprocal interaction of both molecules in human Th17 differentiation.

However, CD5 and CK2 exert differential effects on IFN-γ production and proliferation. While the effect of CK2 inhibition was at least partly mediated by the decreased proliferation of T cells, CD5 blockade led to a proliferation-independent reduction in cytokine production. Moreover, although both treatments resulted in decreased phosphorylation of STAT3, the timeline for this effect differed. It is important to note that CK2 is involved in phosphorylation of up to 356 different proteins.^23^ For example, CK2 partakes in the phosphoinositide 3-kinase (PI3K)/protein kinase B (Akt)/mammalian target of rapamycin complex 1 (mTORC1) pathway and thereby influences cell metabolism and autophagy.^24^ Thus, the CD5-CK2-STAT3 axis represents a major coherent signalling pathway in Th17 differentiation and function, but each of the interaction partners also acts via independent mechanisms.

Chronic neuroinflammation, such as that seen in MS, affects the neuronal compartment including neurogenesis and thereby disturbs higher cerebral functions.^25,26^ For example, T cells producing pro-inflammatory cytokines were previously shown to inhibit proliferation of neural stem cells.^27^ In our study, we found that Th17-polarized cells significantly reduced human neural stem cells in a co-culture model. Importantly, pretreatment of T cells with CD5 blockade abolished their detrimental effect.

In addition to these *in vitro* findings, both our transcriptomic and proteomic analyses showed an association of CD5 expression with pro-inflammatory Th1/Th17 signalling and activation pathways in serum and CSF from people with MS. Activated Th cells have already been described in inflammatory meningeal infiltrates from progressive MS patients, linking their presence to neurodegeneration.^28^ On a transcriptomic level, we were able to compare people with MS to patients with NIND and found more pronounced inflammatory signals linked to CD5 in neuroinflammation. Markers specifically enriched in CD5+ CD4+ T cells from people with MS compared with NIND included, for example granzyme K, which has been described as a marker for brain-homing, pathogenic Th17.1 cells in neuroinflammation, and an integrin-β chain (IGTB2) also involved in immune cell activation.^29–31^ On a protein level, CD5 was previously reported to be increased in CSF from people with MS compared with healthy controls, and to be positively correlated with neurofilament light.^32^ This was in line with our observations in serum in patients outside of relapse activity. Interestingly, CD5 levels were previously demonstrated to decrease upon treatment with fingolimod or natalizumab.^33^ Altogether, we suggest CD5 as an MS biomarker linked to active inflammation and neurodegeneration.

To transfer those findings to clinical practice, one has to consider potential side effects of therapeutic targeting. While being primarily known for its role in T cells, CD5 may also be expressed by other cell types. For example, CD5+ B cells are involved in the production of low-affinity polyreactive antibodies and have been discussed in both autoimmunity and malignant transformation, especially chronic lymphocytic leukaemia.^34,35^ Moreover, a subset of dendritic cells has also been described to express CD5, associated with enhanced functions in T cell priming.^36^ Both B cells and dendritic cells also play a role in the pathology that is mostly in the same direction as pro-inflammatory T cells. Thus, blocking the CD5 pathway on these other immune cells may also be beneficial for MS.

Downstream of CD5, our data support STAT3 as a target for T cell modulation. In psoriasis, another Th17-mediated disease, blockade of STAT3 or its associated Janus kinases have already been discussed as potential treatment options.^37,38^ In MS, higher levels of phosphorylated STAT3 were observed in PBMCs from patients during relapse or after *in vitro* activation.^39,40^ Furthermore, polymorphisms in the corresponding gene locus are associated with enhanced risk of MS.^41^ In considering therapeutically targeting STAT3, its broad signalling function in immune and also non-immune cells needs to be kept in mind.

Overall, our data highlight the significance of the CD5-CK2-STAT3 pathway in pro-inflammatory and pathology-mediating Th17 signalling in humans. Further understanding of specific pathways to modulate pathogenic immune cell subsets, without causing a general compromise of the immune system, may contribute to the development of new therapeutic strategies in autoimmune diseases.

## Supplementary Material

awaf268_Supplementary_Data

## Data Availability

Raw data from human CSF scRNA-seq has been deposited in The National Center for Biotechnology Information (NCBI)’s Gene Expression Omnibus (GEO) under the accession code GSE189998.
