# Supplementary material for: Role of CD5 signalling for pro-inflammatory Th17 response in multiple sclerosis
Source: Brain. 2025 Aug 25;149(1):123–33. doi: 10.1093/brain/awaf268 (PMC12782164; doi:10.1093/brain/awaf268)
Supplement: awaf268_Supplementary_Data [file awaf268_supplementary_data.pdf]

Supplementary methods

Single-cell RNA sequencing (scRNA-seq) data analysis

Raw fastq-sequencing reads were uploaded to BD Targeted Rhapsody Analysis Pipeline (SevenBridgesGenomics) and processed using pipeline v1.9. Downstream analysis of targeted single-cell RNA sequencing was performed in R 4.3.0 and the Seurat 4.3.0.1 toolkit.[1] We used scDblFinder 1.14.0[2] to filter out doublets (in singleModelSplitThres mode, with iter = 2 and knownUse="positive"). 952 of 20658 cells (4.6%) were discarded as doublets. Count data was normalized using scran version 1.28.1[3] and then scaled-and-centered in Seurat in order to perform principle component analysis. For clustering, we determined the number of principle components (PCs) to use by a heuristic that tries out how many clusters result from each number of PCs, then maximizing the number of clusters, while $number\ of\ clusters \leq number\ of\ PCs + 1$. Clustering was performed in Seurat using the k-Nearest-Neighbor-Graph with k set to ln(number of cells) and the Leiden[4] algorithm with resolution = 0.289. The clusters were annotated by ScType v1.0 [5]; summarized into a T cell group that was then subsetted in Seurat based on detection of CD3, CD4 and CD5 expression.

Afterwards we performed differential expression (DE) testing using MAST 1.26.0[6] with a glmer (lme4 version 1.1-34) mixed-model incorporating the patient as the random effect, accounting for the cellular detection rate, with nAGQ = 1 and strictConvergence disabled.

We analyzed Gene Ontology term over-representation in each list of significant DE genes against the background (all detected genes) in ClusterProfiler 4.8.2[7] using the org.Hs.eg.db version 3.17.0 gene annotation and visualized a gene-concept network with enrichplot.

References

1. Hao Y, Hao S, Andersen-Nissen E, et al. Integrated analysis of multimodal single-cell data. *Cell*. Jun 24 2021;184(13):3573-3587.e29. doi:10.1016/j.cell.2021.04.048

2. Germain PL, Lun A, Garcia Meixide C, Macnair W, Robinson MD. Doublet identification in single-cell sequencing data using scDblFinder. *F1000Res*. 2021;10:979. doi:10.12688/f1000research.73600.2

3. Lun AT, Bach K, Marioni JC. Pooling across cells to normalize single-cell RNA sequencing data with many zero counts. *Genome Biol*. Apr 27 2016;17:75. doi:10.1186/s13059-016-0947-7

4. Traag VA, Waltman L, van Eck NJ. From Louvain to Leiden: guaranteeing well-connected communities. *Sci Rep*. Mar 26 2019;9(1):5233. doi:10.1038/s41598-019-41695-z

5. Ianevski A, Giri AK, Aittokallio T. Fully-automated and ultra-fast cell-type identification using specific marker combinations from single-cell transcriptomic data. *Nat Commun*. Mar 10 2022;13(1):1246. doi:10.1038/s41467-022-28803-w

6. Finak G, McDavid A, Yajima M, et al. MAST: a flexible statistical framework for assessing transcriptional changes and characterizing heterogeneity in single-cell RNA sequencing data. *Genome Biol*. Dec 10 2015;16:278. doi:10.1186/s13059-015-0844-5

7. Wu T, Hu E, Xu S, et al. clusterProfiler 4.0: A universal enrichment tool for interpreting omics data. *Innovation (Camb)*. Aug 28 2021;2(3):100141. doi:10.1016/j.xinn.2021.100141

Supplementary table 1. Patient data for Olink® cohort.

	**Patients (n=114)**<br>**Mean or *n* (range or %)**
Age [years]	33.1 (18.0-49.4)
Female sex	70 (61.4)
Time since first symptoms [months]	6.6 (0-56.5)
Relapse occurrence in the past 30 days	72 (63.2)
Annual relapse rate (past 12 months)	1.21 (0-2)
EDSS	1.66 (0-4.5)
Immunomodulatory treatment	0 (0)

Characteristics for patients included in Olink® analysis. EDSS: expanded disability status scale.

Supplementary table 2. Patient data for single-cell RNA sequencing cohort.

	**Mean or *n* (range or %)**	
	**RRMS (n=3)**	**NIND (n=3)**
Age [years]	28 (21-37)	33 (25-42)
Female sex	3 (100)	3 (100)
Time since first symptoms [months]	10.3 (1-18)	*na*
EDSS	1.8 (0-3.5)	*na*
Immunomodulatory treatment	0 (0)	0 (0)
CSF leukocyte count /µl	6 (5-7)	2 (1-3)
CSF protein [mg/dl]	33.1 (28.1-40.1)	32.0 (25.6-38.4)

Characteristics for patients included in single-cell RNA sequencing. RRMS: relapsing-remitting multiple sclerosis; NIND: non-inflammatory neurological disorder; na: not applicable; EDSS: expanded disability status scale; CSF: cerebrospinal fluid.

Supplementary table 3. Olink® panel.

Protein	Exclusion due to call rate < 25%
IL8	
VEGFA	
CD8A	
MCP-3	CSF
GDNF	CSF
CDCP1	
CD244	
IL7	CSF
OPG	
LAP TGF-beta-1	
uPA	
IL6	
IL-17C	CSF
MCP-1	
IL-17A	CSF
CXCL11	
AXIN1	CSF
TRAIL	
IL-20RA	CSF
CXCL9	
CST5	
IL-2RB	CSF
IL-1 alpha	serum
OSM	CSF
IL2	serum, CSF
CXCL1	
TSLP	CSF
CCL4	
CD6	
SCF	
IL18	
SLAMF1	CSF
TGF-alpha	
MCP-4	
CCL11	
TNFSF14	
FGF-23	CSF
IL-10RA	CSF
FGF-5	
MMP-1	
LIF-R	
FGF-21	CSF
CCL19	
IL-15RA	CSF
IL-10RB	
IL-22 RA1	CSF
IL-18R1	
PD-L1	
Beta-NGF	serum, CSF
CXCL5	
TRANCE	CSF
HGF	
IL-12B	
IL-24	serum, CSF
IL13	serum, CSF

ARTN	CSF
MMP-10	
IL10	CSF
TNF	
CCL23	
CD5	
CCL3	
Flt3L	
CXCL6	
CXCL10	
4E-BP1	
IL-20	serum, CSF
SIRT2	
CCL28	
DNER	
EN-RAGE	
CD40	
IL33	serum, CSF
IFN-gamma	
FGF-19	
IL4	CSF
LIF	serum
NRTN	CSF
MCP-2	
CASP-8	
CCL25	
CX3CL1	CSF
TNFRSF9	
NT-3	CSF
TWEAK	
CCL20	CSF
ST1A1	CSF
STAMBP	
IL5	CSF
ADA	
TNFB	CSF
CSF-1	

Supplementary table 4. Differential gene expression using MAST of CD4+CD5+ T cells compared between people with multiple sclerosis (pwMS) and patients with non-inflammatory neurological diseases (NIND).

Primer ID	p-value	Log2 fold change	Confidence interval upper bound	Confidence interval lower bound	Adjusted p-value
DUSP2	4.67E-33	0.413837366	0.495041278	0.332633454	9.15E-31
EGR1	1.04E-32	0.289016011	0.51692151	0.061110512	2.04E-30
CD69	2.26E-21	0.342949827	0.41710214	0.268797514	4.43E-19
IFITM2	2.26E-17	-0.258643859	-0.194419876	-0.322867843	4.43E-15
ITGB2	1.12E-10	0.180716789	0.240267037	0.121166541	2.20E-08
MYC	2.92E-10	-0.238852433	-0.168421493	-0.309283374	5.72E-08
KCNA3	1.68E-09	-0.081014641	-0.047701411	-0.11432787	3.29E-07
RGS1	5.04E-09	0.148318018	0.207386416	0.089249621	9.89E-07
CTSH	1.24E-08	-0.073621473	-0.037645482	-0.109597463	2.44E-06
CST7	1.84E-08	0.186407851	0.25154025	0.121275452	3.61E-06
JUNB	6.43E-08	0.096253078	0.147096497	0.045409658	1.26E-05
PDIA4	9.51E-08	-0.066237986	-0.030766974	-0.101708997	1.86E-05
XBP1	1.60E-07	-0.077200057	-0.018840661	-0.135559452	3.14E-05
FAM65B	9.56E-07	-0.141526052	-0.087437621	-0.195614482	0.000187313
CD14	1.71E-06	-0.012200555	0.008762226	-0.033163336	0.000335328
PDIA6	1.80E-06	-0.099305729	-0.049094074	-0.149517384	0.000352896
GZMK	4.09E-06	0.197262188	0.277762623	0.116761753	0.000801674
LIPA	2.66E-05	0.109907082	0.158614449	0.061199715	0.005211006
IFITM3	3.02E-05	-0.154508348	-0.086728319	-0.222288378	0.005924007
FCER1G	4.02E-05	-0.016073183	0.000497247	-0.032643613	0.00788881
PRDM1	6.08E-05	-0.098051305	-0.045498823	-0.150603787	0.011915136
NAMPT	6.95E-05	-0.037062	-0.017678469	-0.056445531	0.01362813
BCL2A1	0.000136725	-0.027436058	0.001635086	-0.056507203	0.026798122
PRDX5	0.000139082	-0.100942748	-0.050834537	-0.151050959	0.02726006

Supplementary table 5. Differential gene expression using MAST of CD4+CD5- T cells compared between people with multiple sclerosis (pwMS) and patients with non-inflammatory neurological diseases (NIND).

Primer ID	p-value	Log2 fold change	Confidence interval upper bound	Confidence interval lower bound	Adjusted p-value
EGR1	1.46E-14	0.21788717	0.41204153	0.0237328	2.87E-12
CD69	5.17E-12	0.27363082	0.35597525	0.19128639	1.01E-09
DUSP2	1.56E-10	0.24868678	0.32878656	0.16858701	3.05E-08
CTSH	5.71E-09	-0.13353875	-0.08024738	-0.18683011	1.12E-06
XBP1	1.15E-08	-0.15642846	-0.0893356	-0.22352132	2.26E-06
PDIA4	5.80E-07	-0.08839517	-0.04633983	-0.13045052	0.00011377
IFITM2	7.41E-07	-0.17894042	-0.10748892	-0.25039192	0.0001453
MYC	6.05E-06	-0.19120819	-0.11224786	-0.27016852	0.00118574
RGS1	7.50E-06	0.15613679	0.22887934	0.08339425	0.00146927
PRDX5	1.59E-05	-0.06512859	-0.0078015	-0.12245569	0.00311107
CD8A	0.00020084	-0.05159648	-0.02449013	-0.07870283	0.0393641

Supplementary table 6. Differential gene expression using MAST of CD4+CD5+ T cells compared to CD4+CD5- T cells in people with multiple sclerosis (pwMS).

Primer ID	p-value	Log2 fold change	Confidence interval upper bound	Confidence interval lower bound	Adjusted p-value
CD6	9.09E-48	0.418330114	0.475667209	0.36099302	1.75E-45
TRAC	8.85E-12	0.216417342	0.278529299	0.154305385	1.70E-09
LEF1	1.94E-10	0.193503614	0.250156536	0.136850691	3.73E-08
CCR7	2.67E-10	0.172852549	0.224177101	0.121527998	5.13E-08
CD3E	6.64E-09	0.157251352	0.213678884	0.10082382	1.28E-06
TRAT1	1.38E-07	0.137196574	0.184871895	0.089521253	2.64E-05
LAT	6.36E-07	0.125180008	0.176373617	0.073986399	0.000122092
CD52	7.12E-07	0.153212279	0.210117903	0.096306655	0.000136777
LCK	8.23E-07	0.136647857	0.188573942	0.084721773	0.000158048
TRBC2	1.13E-06	0.234934883	0.324722901	0.145146865	0.000216927
CD2	9.55E-06	0.101784188	0.160103845	0.04346453	0.00183315
CD3D	1.04E-05	0.125268034	0.177545759	0.072990309	0.001991944
DUSP1	2.05E-05	-0.123685905	-0.067408455	-0.179963354	0.003939601
CD74	2.94E-05	-0.2206567	-0.114819112	-0.326494287	0.00564279
CD63	4.09E-05	-0.1210368	-0.066380456	-0.175693144	0.007861005
IL32	4.78E-05	0.141332519	0.206825453	0.075839585	0.00918143
HLA.A	4.88E-05	0.089411665	0.128712759	0.05011057	0.009373006
GIMAP5	0.000147326	0.116537807	0.172228646	0.060846969	0.028286668
BCL11B	0.000190534	0.086430821	0.127595983	0.045265659	0.036582556
TRIB2	0.000241606	0.102833799	0.152509381	0.053158218	0.046388287

Supplementary table 7. Differential protein expression in CD5 high versus CD5 low in serum.

Protein	p-value	adjusted p-value	minus log10(p)	mean of CD5 high group	mean of CD5 low group	fold change	log2 fold change
TNFRSF9	1.19986E-08	9.95884E-07	6.00179145	7.151968125	6.148467059	2.004859402	1.003501066
CD6	8.70755E-07	7.14019E-05	4.146290018	6.72167625	5.635082353	2.123720485	1.086593897
SLAMF1	1.17243E-06	9.49672E-05	4.022426462	2.7622425	2.194217647	1.482492548	0.568024853
TRAIL	3.56016E-06	0.000284812	3.545441099	8.019690625	7.319293529	1.624951992	0.700397096
CCL25	7.6054E-06	0.000600826	3.221251062	6.2762825	5.462372941	1.757968913	0.813909559
CD244	7.6054E-06	0.000600826	3.221251062	6.698189375	6.095274706	1.518781856	0.602914669
IL-17C	7.6054E-06	0.000600826	3.221251062	2.593413125	1.699904706	1.857688256	0.893508419
IL-12B	1.93023E-05	0.001466976	2.833577032	6.569498125	5.570303529	1.998883784	0.999194596
IL-10RB	3.6882E-05	0.002766148	2.558124599	6.54525	6.013264118	1.445918145	0.531985882
uPA	3.6882E-05	0.002766148	2.558124599	10.29653	9.722148235	1.489039231	0.574381765
PD-L1	4.53821E-05	0.003312895	2.47979235	5.865595625	5.323648235	1.455936455	0.54194739
LIF-R	0.000120672	0.008688355	2.061062427	4.635865625	4.237118824	1.318362217	0.398746801
CDCP1	0.000174079	0.012359614	1.907995094	2.568019375	2.009585882	1.472669295	0.558433493
MMP-10	0.000174079	0.012359614	1.907995094	9.6165825	8.757862353	1.813428856	0.858720147
TRANCE	0.000174079	0.012359614	1.907995094	5.233421875	4.366154118	1.824204863	0.867267757
CSF-1	0.00034877	0.023716365	1.624951881	10.66984	10.35198706	1.24647413	0.317852941
IL-17A	0.000411896	0.027597059	1.559137202	1.767411875	1.260721176	1.420787406	0.506690699
CD8A	0.000569669	0.037598179	1.424833191	9.514548125	8.421111765	2.133816862	1.09343636
MCP-3	0.000569669	0.037598179	1.424833191	2.8758875	1.819555294	2.079637688	1.056332206
IL-15RA	0.000779499	0.049887944	1.302004396	1.370166875	1.031199412	1.264851016	0.338967463
IL18	0.000779499	0.049887944	1.302004396	9.04457125	8.252507647	1.731549467	0.792063603

Supplementary table 8. Differential protein expression in CD5 high versus CD5 low in cerebrospinal fluid (CSF).

Protein	p-value	adjusted p-value	minus log10(p)	mean of CD5 high group	mean of CD5 low group	fold change	log2 fold change
CD6	1.90105E-12	1.12162E-10	9.95015416	3.017660476	1.132585455	3.693721315	1.885075022
CD8A	5.70315E-11	3.2508E-09	8.488010061	5.074362381	2.883471818	4.565872466	2.190890563
CASP-8	8.196E-08	4.58976E-06	5.3382099	2.054616667	1.162403182	1.85602158	0.892213485
TNFSF14	1.00607E-07	5.53341E-06	5.257007233	2.601636667	1.655999545	1.926039274	0.945637121
CD244	2.54869E-06	0.000137629	3.861289548	1.614604286	1.173605909	1.357543452	0.440998377
LAP TGF-beta-1	3.88629E-05	0.002059734	2.686188881	3.79537	2.315152273	2.789908346	1.480217727
TNFB	4.70508E-05	0.002446641	2.611429705	3.008042857	2.016608182	1.988161117	0.991434675
IL-12B	7.29302E-05	0.003719441	2.42952238	4.221713333	2.610293636	3.055523754	1.611419697
TNFRSF9	7.29302E-05	0.003719441	2.42952238	3.795520476	3.000842727	1.734689858	0.794677749
4E-BP1	8.24005E-05	0.004037625	2.393873969	1.588198095	1.023103182	1.47948484	0.565094913
CXCL11	0.000104805	0.005030656	2.298375413	3.163750476	1.822007727	2.534573057	1.341742749
IFN-gamma	0.000187315	0.008803807	2.055329468	5.49628	3.741046818	3.37580877	1.755233182
IL-18R1	0.000292117	0.013437386	1.87168521	3.370808095	2.812589091	1.472450366	0.558219004
CXCL1	0.000362458	0.016310623	1.787529438	5.422522381	4.41774	2.006640788	1.004782381
STAMBP	0.000362458	0.016310623	1.787529438	0.849985714	0.578108636	1.207377715	0.271877078
TRAIL	0.0004971	0.021375299	1.670087811	1.19755	0.778402727	1.337136986	0.419147273
CXCL10	0.000746938	0.031371405	1.503466025	8.838843333	6.586509091	4.764531112	2.252334242

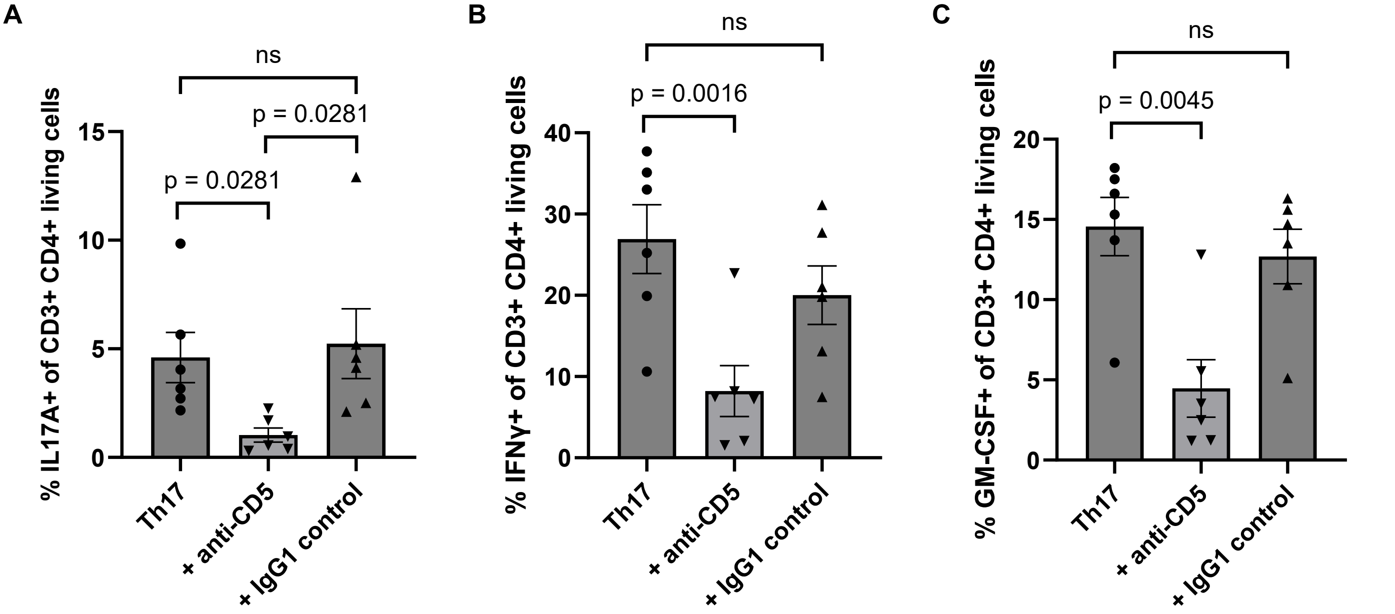

Supplementary Figure 1. Treatment of Th17-polarized cultures with unspecific IgG1 control. Treatment of Th17-polarized cells with an unspecific IgG1 antibody did not reduce production of cytokines, in contrast to CD5 blockade. Data shown for (**A**) IL-17A, (**B**) IFNγ and (**C**) GM-CSF. Flow cytometric data for n=6 cultures, each culture represents a unique human donor. Statistical analysis was performed by Friedman test with correction for multiple comparisons.

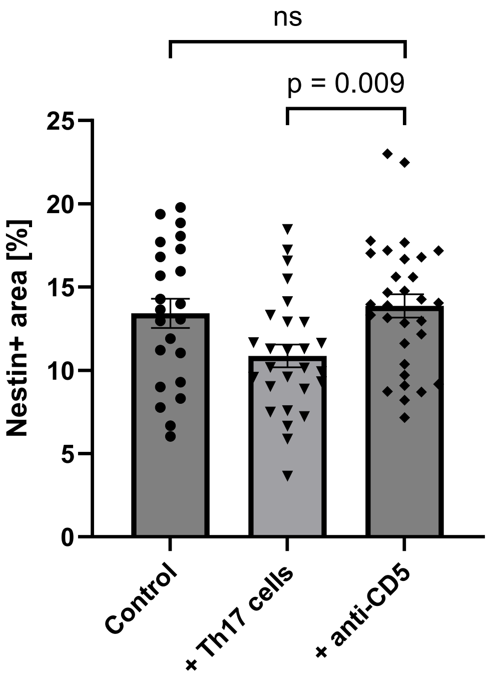

Supplementary Figure 2. Morphological analysis of human neural cells. Analysis of nestin+ area (shown as % nestin+ staining area per FOV) in NSC-Th17 cocultures shows a preserved morphology in NSCs cocultured with anti-CD5 treated Th17-polarized cells relative to control while a reduction of nestin+ area (coinciding with a total reduction of nestin+ cells as shown in main Figure 3) was detectable in Th17-polarized cells without CD5 blockade. Statistical analysis was performed by Tukey's multiple comparisons test. Pooled data from two independent experiments. Each experiment represents a unique human donor.

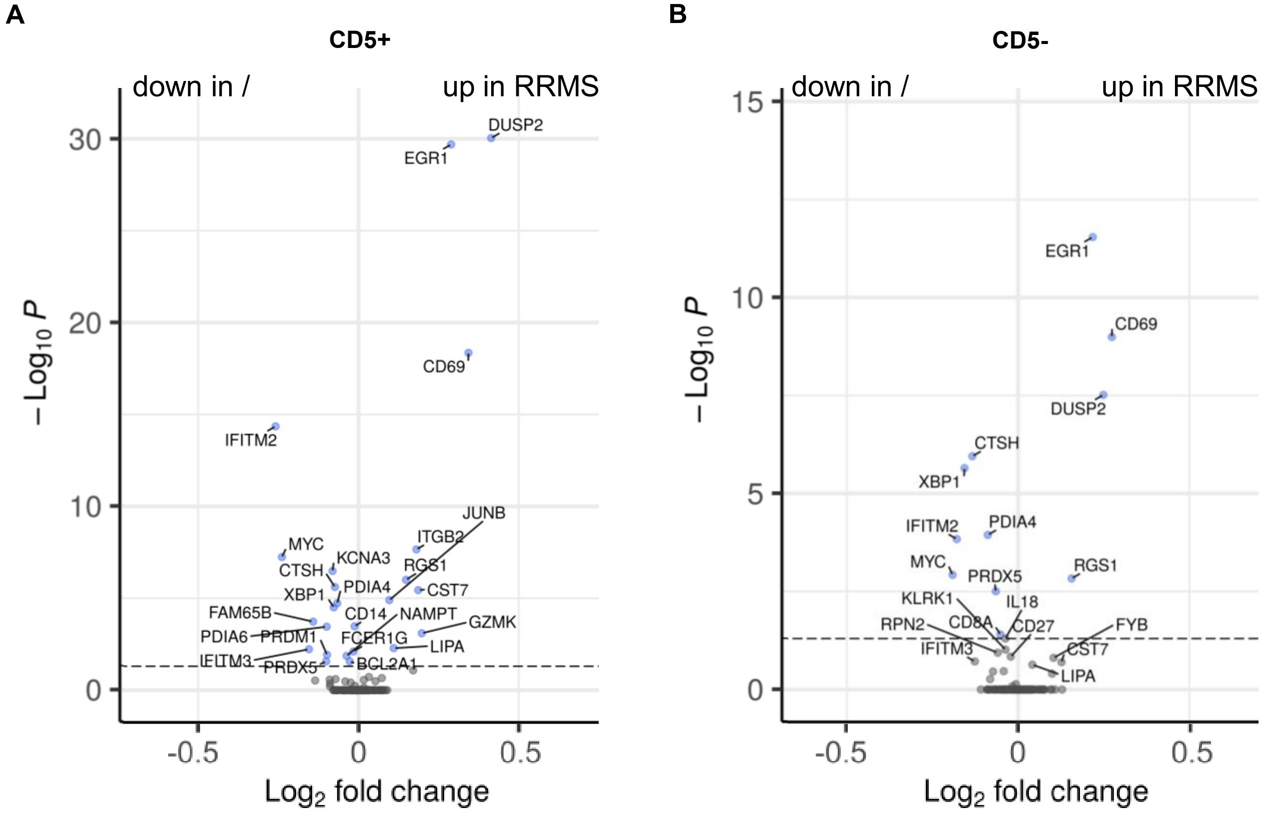

Supplementary Figure 3. Analysis of CD5+ and CD5- CD4+ T cells in cerebrospinal fluid (CSF) by targeted scRNA-seq. (**A**) Mixed-effect MAST differential gene expression (DGE) testing RRMS vs NIND inside the subset of CD5+ CD4+ T cells. Volcano plot of DGE-results. The x-axis represents the point estimates for the $\log_2$ fold changes and the y-axis shows the Bonferroni-adjusted p-values with the significance threshold of 0.05 marked by a dashed line. Image created with R package EnhancedVolcano. (**B**) Mixed-effect MAST differential gene expression (DGE) testing RRMS vs NIND inside the subset of CD5- CD4+ T cells. Volcano plot of DGE-results. The x-axis represents the point estimates for the $\log_2$ fold changes and the y-axis shows the Bonferroni-adjusted p-values with the significance threshold of 0.05 marked by a dashed line. Image created with R package EnhancedVolcano.
